# Short-term prognostic value of TAPSE, RVFAC and Tricuspid S’ wave peak systolic velocity after first acute myocardial infarction

**DOI:** 10.1186/s13104-020-05040-2

**Published:** 2020-04-01

**Authors:** Engy Magdy Lewis Awad, Adel Hamdy Mahmoud, Khaled Sayed Maghrby, Nasser Mohamad Taha, Alaa Mohamed Ibrahim

**Affiliations:** Cardiology Department, Minia University, El-Minia, Egypt

**Keywords:** RVFAC, TAPSE, S’ wave peak systolic velocity, AMI, NSTEMI, Short-term prognosis

## Abstract

**Objectives:**

Right ventricular dysfunction impacts the prognosis of various heart diseases. We set-out to examine which right ventricular functional parameters after STEMI and NSTEMI have prognostic value. Of 297 eligible participants, 266 (149 STEMI and 117 NSTEMI) completed follow-up. All patients underwent Grace score and 2D-echocardiography within 24 h. Outcome was defined as occurrence of Major Adverse Cardiovascular events (MACE), such as death, recurrent ischaemia, arrhythmia, reinfarction, stroke or heart failure, within 30 days. Patients were categorized into patients with MACE and patients without MACE.

**Results:**

In STEMI-patients, compared to those without MACE, patients with MACE experienced higher grace score, left ventricle (LV) end-systolic volume, LV end-systolic dimension and wall motion score index values, but lower tricuspid annular plane systolic excursion, right ventricle (RV) fractional area change, Tricuspid S’ wave peak systolic velocity and LV ejection fraction. Nevertheless, in NSTEMI-patients, those with MACE exhibited higher left atrial volume index values, but lower tricuspid annular plane systolic excursion, RV fractional area change, S’ wave peak systolic velocity and LVEF. Right ventricular fractional area change < 37.5%, tricuspid annular plane systolic excursion < 15.8 mm and Tricuspid S’ peak systolic velocity < 9.67 cm/s are independent predictors of MACE within first 30 days after STEMI and NSTEMI.

## Introduction

Compared to the conclusive data on the role of LV in various heart disease, the available information regarding the RV function and its role in heart diseases particularly acute myocardial infarction (AMI), is limited but outlined its significant contribution [1]. A link between right ventricular dysfunction and the prognosis of various heart disease such as heart failure and pulmonary hypertension, was reported [1]. It has been postulated that events in one ventricle affect the contralateral ventricle. Moreover, common pericardium, the in-series circulation, interventricular septum and myocardial tracts running between the ventricles can explain the physiology of ventricular–ventricular interactions [2].

The association between right ventricular functional parameters and STEMI [3–7], but not NSTEMI, was previously examined. The association between the RV function and the outcome in patients with STEMI and NSTEMI is an issue that needs to be addressed in a large cohort of patients. Therefore, we set out to examine the RV functional parameters of acute STEMI or NSTEMI, within the first 24 h after the event. The relationship between those parameters and the prognosis after AMI was also investigated.

## Main text

### Patients and methods

This is a prospective non-randomized study. Approval was obtained from hospital ethics committee. Patients who were admitted with AMI to our CCU from April 2014 to December 2016 were eligible to be enrolled. Consent was obtained from eligible participants. Those with co-morbidities such as renal failure, hepatic failure, cor-pulmonale, dilated cardiomyopathy, connective tissue diseases, pulmonary hypertension, pulmonary embolism, previous MI, permanent pacemaker or right ventricular infarction were excluded.

Of 297 patients, 266 (89.56%) completed the follow up, of whom 117 (43.98%) had NSTEMI and 149 (56.02%) experienced STEMI. In patients with STEMI, 83 of 149 (55.70%) and 66 of 149 (44.30%) had anterior AMI and inferior AMI, respectively. All participants underwent clinical examination (HR, ABP, Killip classification) and blood tests including plasma creatinine, blood glucose, and serum hemoglobin values. Grace risk score was calculated. Echocardiography and tissue Doppler were performed, within the first 24 h of the event, using Vivid 3 (Vingmed, General Electric, Horton, Norway). Data acquisition was performed at a depth of 16 cm in the parasternal and apical views using a 3.5-MHz transducer.

The LV end-systolic volume (LV ESV) and end-diastolic volume (LV EDV) were assessed by biplane Simpson method from the apical 4- and 2-chamber views tracing the LV endocardial-blood pool interface at end-systole and end-diastole. Echocardiographic measurements were performed by expert blinded operator. LV ejection fraction (LVEF) was calculated (EF = EDV − ESV/EDV), using the biplane Simpson method. Tricuspid annular plane systolic excursion (TAPSE) was measured in the M-mode view, in the RV free wall as total displacement of the RV base from end-diastole to end-systole. RVFAC was obtained by tracing the RV end-diastolic area (RVEDA) and end-systolic area (RVESA) in the apical 4-chamber view using the formula (RVEDA − RVESA)/RVEDA × 100. RV basal diameter is the maximal transverse dimension in the basal one-third of RV inflow at end-diastole in the RV-focused view. Tricuspid S’ peak systolic velocity: pulsed-wave DTI at tricuspid annulus, obtained from the apical approach with parallel alignment of Doppler beam with RV free wall longitudinal excursion. LAVI was performed using the LA areas and lengths through tracing of LA endocardial borders in both the apical four- and two-chamber views. As LAV is dependent on gender, indexing to BSA was performed to abolish confounding.

Patients were followed up for 30 days after AMI event and adverse outcome was defined as occurrence any of the major adverse cardiovascular events (MACE) events during the follow up. MACE events include death, hospitalization for recurrent ischemia, reinfarction, hospitalization for heart failure, arrhythmias requiring hospital management, ischemic stroke. Patients were classified into “patients with MACE” group (patients who experienced any of the MACE events within first 30-days period after AMI) and “patients without MACE” group.

Statistical methods: The sample size was calculated with margin of error accepted at 5% and a degree of confidence of 95%. The population size was considered infinite and the response distribution was set at 25%. All statistical calculations were performed using SPSS 22. Descriptive data was presented as mean and Standard Deviation (SD). P value < 0.05 was considered significant. Student t-test was used to compare the mean of clinical, laboratory and doppler variables between the groups. Variable which were significant by t-test, were examined as predictors of the outcome, by using univariate regression analysis. This was followed by multivariate logistic regression analysis to determine the independent predictors of the outcome. Receiver operator characteristic analysis was used to determine the cutoff values for TAPSE, RVFAC and S’ peak systolic velocity.

### Results

In total 266 patients were examined, mean (SD) age 61.19 (7.97) years. Of these 266 individuals, 181 (68%) were male, 79 (29.7%) have diabetes mellitus, 74 (7.2%) were smokers, 66 (24.8%) experienced hypertension and 19 (7.1%) exhibited positive family history of sudden cardiac death. The difference between patients with MACE and those without MACE in terms of clinical and laboratory data, Grace risk assessment score and doppler parameters is illustrated in Tables 1, 2.

**Table 1:** Comparison between patients with MACE and patients without MACE regarding clinical and laboratory data and Grace risk assessment score on admission

	Patients with MACE [n = 106, (39.8%)]	Patients without MACE [n = 160, (60.2%)]	P value*
	Mean (SD)	Mean (SD)	
Age (years)	61.36 (6.941)	61.08 (8.60)	0.78
Heart rate (Bpm)	85.98 (21.43)	88.44 (18.09)	0.32
Systolic blood pressure (mmHg)	127.36 (26.69)	129.438 (23.54)	0.51
Serum creatinine (mg/dl)	1.19 (0.38)	1.24 (0.43)	0.32
Random blood sugar (mg/dl)	203.09 (90.38)	200.43 (85.50)	0.81
Hemoglobin (gm/dl)	12.06 (1.40)	12.13 (1.41)	0.71
Grace score	135.38 (37.49)	126.14 (33.20)	0.036*

Patients with MACE: Patients who died or have experienced events in whole 30-days period post AMI; Patients without MACE: Patients who survived and have not experience events in whole 30-days period post AMI

* p value < 0.05

**Table 2:** Comparison between patients with MACE, patients without MACE regarding echocardiography or tissue Doppler parameters

	Patients with MACE [n = 106, (39.8%)]	Patients without MACE [n = 160, (60.2%)]	(P value)*
	Mean (SD)	Mean (SD)	
RVBD	3.42 (0.47)	3.41 (0.44)	0.88
RVLD	7.82 (0.23)	7.82 (0.23)	0.89
WMSI	7.16 (3.18)	6.68 (2.64)	0.19
Mitral E/A ratio	0.98 (0.34)	1.02 (0.35)	0.49
Mitral E/e′ ratio	11.32 (4.23)	11.28 (3.18)	0.92
LVEF%	38.03 (2.95)	40.09 (3.68)	0.001*
LAVI	28.11 (2.02)	27.64 (1.73)	0.042*
Tricuspid E/A ratio	1.07 (0.34)	1.05 (0.36)	0.67
Tricuspid E/e′ ratio	6.73 (2.45)	6.44 (1.95)	0.29
S’ wave PTSV (cm/s)	10.18 (1.79)	11.39 (2.17)	0.001*
TAPSE	1.52 (0.13)	1.70 (0.17)	0.001*
RVFAC%	36.41 (2.17)	39.27 (2.97)	0.001*
LV ESd (mm)	29.56 (2.27)	29.03 (2.20)	0.06
LV EDd (mm)	48.58 (2.05)	48.65 (2.08)	0.79
LV EDV (ml)	147.29 (12.44)	144.43 (11.86)	0.06
LV ESV (ml)	46.66 (6.34)	44.57 (6.33)	0.009*

Patients with MACE: Patients who have experienced MACE events within 30-days period after AMI. Patients without MACE: Patients who have not experience events in whole 30-days period post AMI. LVEF%: left ventricular ejection fraction; LAVI: left atrial volume index; RVFAC: right ventricular fractional area change; LV ESd: left ventricular end-systolic diameter; LV EDd left ventricular end-diastolic diameter; LV EDV (left ventricular end-diastolic volume, LV ESV: left ventricular end-systolic volume. RVLD: RV longitudinal diameter; RVBD: Rt ventricular basal diameter; S’ wave PTSV: S’ wave peak tricuspid systolic velocity; TAPSE: tricuspid annular plane systolic excursion; WMSI: wall motion score index

* p value < 0.05

In these 266 patients, univariate regression analysis showed a significant association between MACE and LVEF, LAVI, TAPSE, RVFAC, S’ wave peak systolic velocity, LV ESV and Grace score (p < 0.05). However, by using multivariate regression, LVEF (p value = 0.032), TAPSE (p = 0.009), RVFAC (p = 0.012) and S’ wave peak systolic velocity (p = 0.001) remained significant as independent predictor for the adverse outcomes.

Of 266 patients, 149 (56.02%) had STEMI and 117 (43.98%) experienced NSTEMI. In those with STEMI, MACE occurred in 51 of 149 (34.22%) patients; arrythmia required admission [7 (4.70%)], hospitalization for recurrent ischemia [5 (3.35%)], recurrent infarction [4 (2.68%), hospitalization for heart failure [6 (4.03%)], cardiogenic shock [4 (2.68%)], stroke [2 (1.34%)] and death [23 (15.44%)]. However, in those with NSTEMI, MACE events were reported in 55 of 117 (47%); arrythmia require hospital management [11 (9.4%)], hospitalization for recurrent ischemia [7 (6%)], reinfarction [6 (5.1%)], hospitalization for heart failure [5 (4.3%)], cardiogenic shock [5 (4.3%)] and death [21 (17.9%)].

Table 3 showed that in patient with STEMI, TAPSE, RVFAC, S’ wave peak systolic velocity, LVEF values, LV ESV, LV ESd, WM score index and Grace score were significantly different between individuals with MACE and those without MACE. However, in those with NSTEMI, only LAVI, TAPSE, RVFAC, S’ wave peak systolic velocity and LVEF values were significantly different between patients with and without MACE.

**Table 3:** Mean (SD) of Grace score, echocardiography parameters and tissue doppler values between the two groups of patients (patients with MACE and patients without MACE) in patients with STEMI and NSTEMI

	STEMI (n = 149)			NSTEMI (n = 117)		
	Patients with MACE	Patients without MACE	P value^§^	Patients with MACE	Patients without MACE	P value^§^
	[mean (SD)]	[mean (SD)]		[mean (SD)]	[mean (SD)]	
Grace score	140.27 (41.46)	123.81 (34.10)	0.01^§^	130.84 (33.13)	129.82 (31.65)	0.87
Mitral E/A ratio	0.97 (0.32)	0.99 (0.36)	0.72	0.99 (0.37)	1.06 (0.33)	0.41
Mitral E/e’ ratio	11.23 (4.35)	11.37 (3.24)	0.85	11.40 (4.15)	11.16 (3.10)	0.72
Tricuspid E/A	1.08 (0.33)	1.03 (0.34)	0.38	1.06 (0.33)	1.08 (0.38)	0.69
Tricuspid E/e’	6.96 (2.94)	6.55 (1.91)	0.30	6.51 (1.87)	6.27 (2.01)	0.50
RVBD	3.41 (0.50)	3.44 (0.43)	0.64	3.42 (0.43)	3.35 (0.46)	0.37
RVLD	7.76 (0.21)	7.81 (0.24)	0.21	7.88 (0.23)	7.89 (0.23)	0.51
WMSI	8.61 (2.85)	7.30 (2.53)	0.005^§^	5.81 (2.88)	5.71 (2.51)	0.84
TAPSE (cm)	1.50 (0.12)	1.68 (0.16)	0.001^§^	1.53 (0.14)	1.72 (0.18)	0.001^§^
S’ wave PTSV (cm/s)	9.80 (1.21)	11.12 (2.04)	0.002^§^	10.52 (2.15)	11.81 (2.32)	0.003^§^
RVFAC (%)	36.48 (2.15)	39.03 (2.91)	0.001^§^	36.35 (2.21)	39.65 (3.06)	0.005^§^
LV EF (%)	38.18 (3.04)	40.23 (3.77)	0.02^§^	37.90 (2.88)	39.80 (3.55)	0.002^§^
LAVI	28.25 (2.20)	27.85 (1.66)	0.21	27.99 (1.87)	27.31 (1.80)	0.041^§^
LV ESd (mm)	29.92 (2.05)	28.97 (2.24)	0.013^§^	29.23 (2.43)	29.14 (2.14)	0.82
LV EDd (mm)	48.91 (2.04)	48.67 (2.07)	0.501	48.28 (2.03)	48.62 (2.13)	0.37
LV EDV (ml)	147.22 (11.59)	143.24 (11.76)	0.051	147.36 (13.2)	146.32 (11.87)	0.66
LV ESV (ml)	46.58 (5.64)	44.51 (6.38)	0.045^§^	46.73 (6.98)	44.66 (6.30)	0.09

LAVI: left atrial volume index; LV EF: left ventricular ejection fraction; RVBD: Rt ventricular basal diameter; RVFAC: Right ventricular fractional area change; RVLD: RV longitudinal diameter; S’ wave PTSV: S’ peak tricuspid systolic velocity; TAPSE: Tricuspid annular plane systolic excursion; WMSI: wall motion score index

^§^p value < 0.05

The link between MACE events and echocardiography parameters and doppler values was examined. In patients with STEMI, by using univariate regression, MACE was significantly associated with Grace score, TAPSE, S’ wave peak systolic velocity, RVFAC, LVEF, LV ESV, LV ESd and WMSI (Wall Motion Score Index) (p value < 0.05). However, by using multivariate regression analysis, only TAPSE, S’ wave peak systolic velocity and RVFAC remained significant (p value < 0.05). Similarly, in those with NSTEMI, using univariate regression analysis showed that there was a significant association between the adverse outcome and LVEF, TAPSE, RVFAC, S’ wave peak systolic velocity and LAVI (p value < 0.05), notably this association was not documented with Grace score, LV ESV, LVESd or WMSI (p value > 0.05). However, by using multivariate regression analysis, LVEF (p value 0.041), TAPSE (p = 0.001), RVFAC (p = 0.001) and S’ wave peak systolic velocity (p = 0.008) remained significant as independent predictor for the adverse outcomes.

By the end of the first 30 days post AMI, the overall MACE events were noticed in 64.8% of patients who have RV dysfunction (n = 54) (defined as RVFAC < 35%), but only in 33.5% of patients with normal RV function (n = 212) post AMI [odds ratio 3.65 (95% CI 1.95–6.85), P < 0.001].

Receiver operator characteristic (ROC) analysis was used to examine the cutoff of various variables and their sensitivity and specificity in predicting the adverse outcome. We have noted that the TAPSE cutoff value was 15.8 mm, with area under the curve (AUC) 0.803, 95% CI (0.749–0.859), sensitivity of 79.3% and specificity 75.1%. The cutoff value for RVFAC was 37.5%, with AUC 0.783), 95% CI (0.727–0.839), sensitivity 72.6% and specificity 73.7%. S’ peak systolic velocity cutoff value was 9.67 cm/s, AUC 0.783, 95% CI (0.670–0.797), 73% sensitivity and 65.0% specificity), (Additional file 1: Fig S1).

### Discussion

In the current study, we have demonstrated the right ventricular functional parameters within the first 24 h from AMI and their relation to MACE in those presented with STEMI or NSTEMI. In STEMI and NSTEMI patients; a link has been observed between RV dysfunctional parameters such as RVFAC, TAPSE and S’ peak systolic velocity and occurrence of MACE events within the first 30 days after AMI. In patients with STEMI and NSTEMI, individuals with MACE had lower TAPSE, RVFAC, S’ wave peak systolic velocity and LVEF values than those without MACE. However, they experienced higher Grace score, LVESd, LVESV and WMSI values, in patients with STEMI, and higher LAVI values in those with NSTEMI.

Emerging evidence suggests that the right ventricular dysfunction is an important predictor of cardiac events and mortality in multiple cardiac diseases [1, 8]. In this cohort of patients, RV dysfunction parameters such as TAPSE, RVFAC, and S’ wave peak systolic velocity of the tricuspid valve were independent predictors for MACE outcome within first 30 days after acute AMI.

In this cohort of patients, we have found that right ventricular dysfunction occurs in individuals with inferior as well as anterior infarction, in accordance with previous studies [3, 4, 9]. It was suggested that LAD occlusion results in right ventricular dysfunction through impaired septal contractility as a result of decreased supply through septal perforators and ischemia of posterior aspect of apical septum [3]. Moreover, impaired RV function could occur through increased afterload after LV dysfunction from anterior infarction [3]. In patients studied with Cardiac Magnetic Resonance, right ventricular ischemic changes were found adjacent to the ischemic dysfunctional left ventricular myocardium [5]. Moreover, this pattern was observed in inferior left ventricular infarcts as well as in up to 33% of anterior left ventricular infarcts, with an impact on right ventricular performance [5].

We noted a link between MACE event and RV dysfunctional parameters such as RVFAC and TAPSE. Similarly, the association between RVFAC or TAPSE and adverse outcome has been reported in previous study, in patients with AMI [7]. In patients with acute inferior STEMI, an association between TAPSE value ≤ 14 mm and adverse outcome was demonstrated in previous study [10]. However, Lohitashwa and colleagues have been shown that RVFAC, but not TAPSE, was associated with adverse outcome in setting of STEMI [6]. Moreover, in STEMI patients, particularly those with impaired left ventricular function in the context of anterior MI, a link between right ventricular dysfunction and ventricular tachycardia has been suggested [3]. Furthermore, the severity of right ventricular dysfunction after AMI has significant impact on long-term mortality [11].

In this cohort of patients, TAPSE and RVFAC are independent predictors for MACE after STEMI, in accordance with the previous studies [4, 10]. Interestingly, we have noted that TAPSE and RVFAC are independent predictors for MACE after NSTEMI as well. In addition, Tricuspid S’ wave peak systolic velocity was independent predictor of MACE within first 30 days after both STEMI and NSTEMI.

### Conclusion

Compared to patients without MACE, patients with MACE had lower TAPSE, RVFAC, S’ wave peak systolic velocity and LVEF values, in patients with STEMI and NSTEMI. However, they experienced higher Grace score, LVESV, LVESd and WMSI values in patients with STEMI, and higher LAVI values in those with NSTEMI.

In STEMI and NSTEMI patients; RV dysfunctional parameters in terms of RVFAC < 37.5%, TAPSE < 15.8 mm and S’ peak systolic velocity < 9.67 cm/s are independent predictors of MACE events within the first 30 days after AMI. These parameters should be used in clinical settings to identify those with high risk.

## Limitation

Single center-based study.

Data regarding the invasive catheterization was not available for all patients.

## Supplementary information

**Additional file 1:** Fig. S1. ROC curve for TAPSE, RVFAC, S’ peak systolic velocity.

## Data Availability

The datasets used and/or analyzed during the current study are available from the corresponding author on reasonable request.

## Abbreviations

AMI: Acute myocardial infarction
DTI: Doppler tissue imaging
MACE: Major adverse cardiovascular events
LAD: Left anterior descending
LA: Left atrium
LAVI: Left atrial volume index
LV: Left ventricle
LV EDd: Left ventricular end-diastolic diameter
LV EDV: Left ventricular end-diastolic volume
LVEF: Left ventricular ejection fraction
LV ESd: Left ventricular end-systolic diameter
LV ESV: Left ventricular end-systolic volume
NSTEMI: Non-ST segment elevation myocardial infarction
RCA: Right coronary artery
RV: Right ventricle
RVBD: Rt ventricular basal diameter
RVEDA: Right ventricular end diastolic area
RVESA: Right ventricular end-systolic area
RVFAC: Right ventricular fractional area change
RVLD: RV longitudinal diameter
S’ wave PTSV: S’ wave peak tricuspid systolic velocity
SD: Standard deviation
STEMI: ST segment elevation myocardial infarction
TAPSE: Tricuspid annular plane systolic excursion
WMSI: Wall motion score index
