# Supplementary figures and images for: Short-term prognostic value of TAPSE, RVFAC and Tricuspid S’ wave peak systolic velocity after first acute myocardial infarction
Source: BMC Res Notes. 2020 Apr 1;13:196. doi: 10.1186/s13104-020-05040-2 (PMC7333336; doi:10.1186/s13104-020-05040-2)

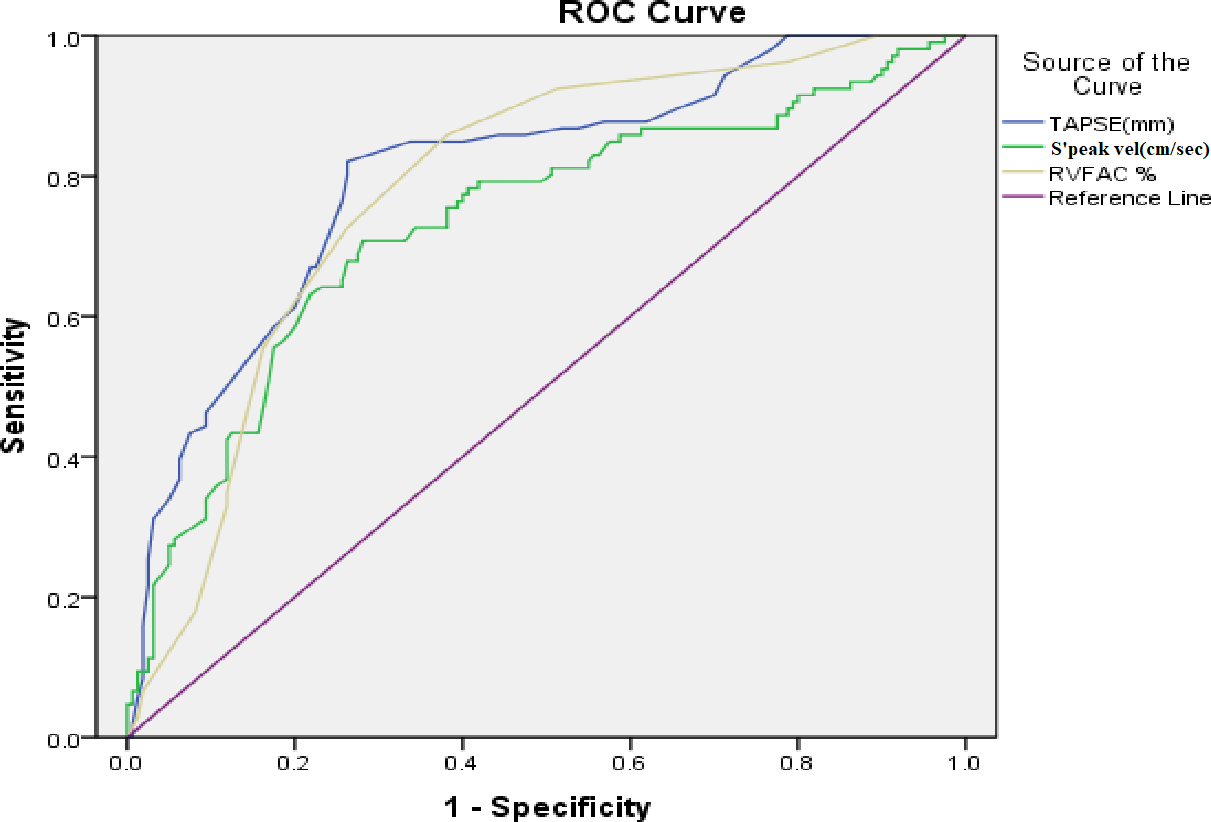

Supplement: Supplementary file 1 — Additional file 1: Fig. S1. ROC curve for TAPSE, RVFAC, S’ peak systolic velocity. [file 13104_2020_5040_MOESM1_ESM.tif]
